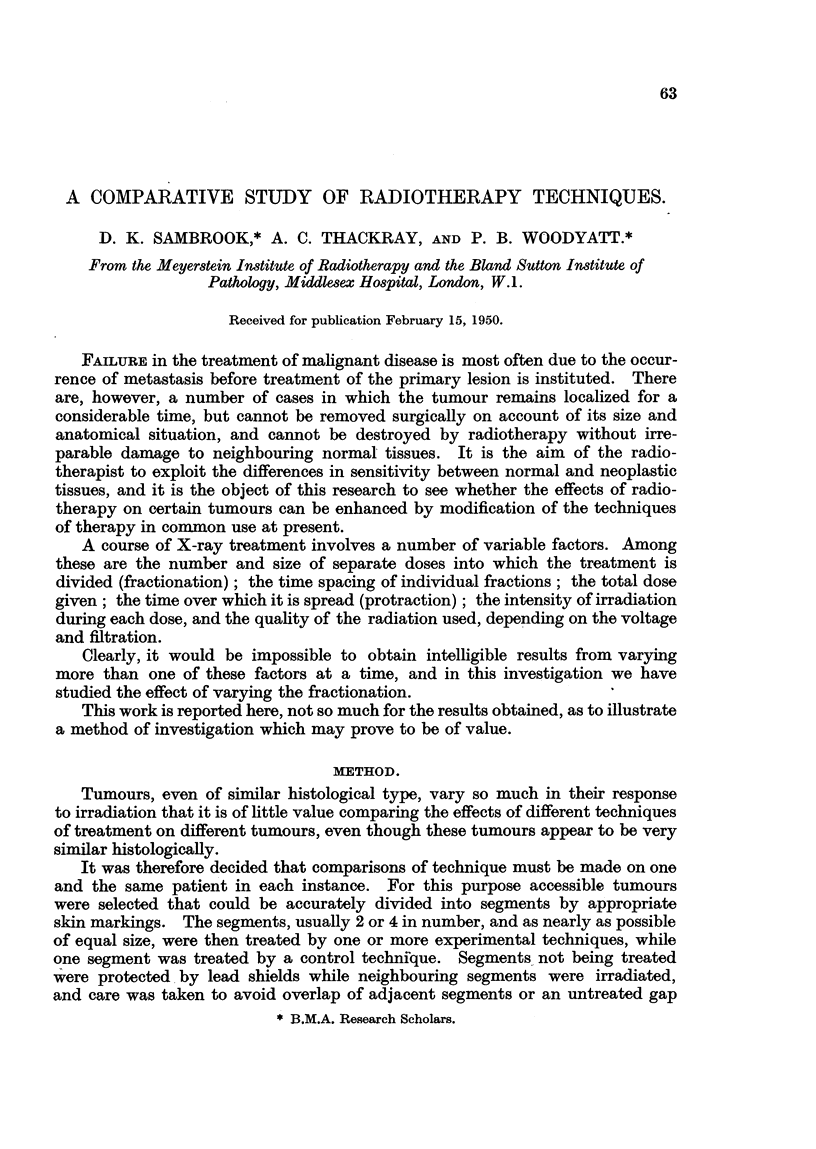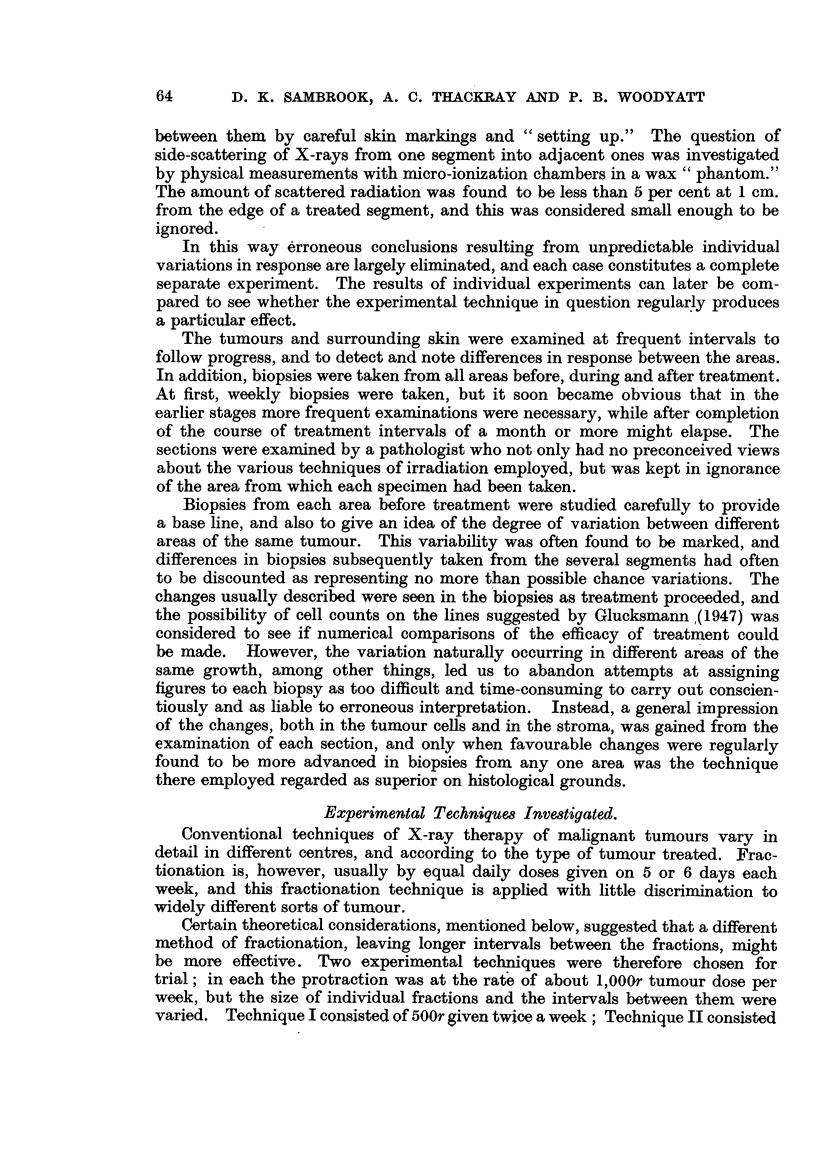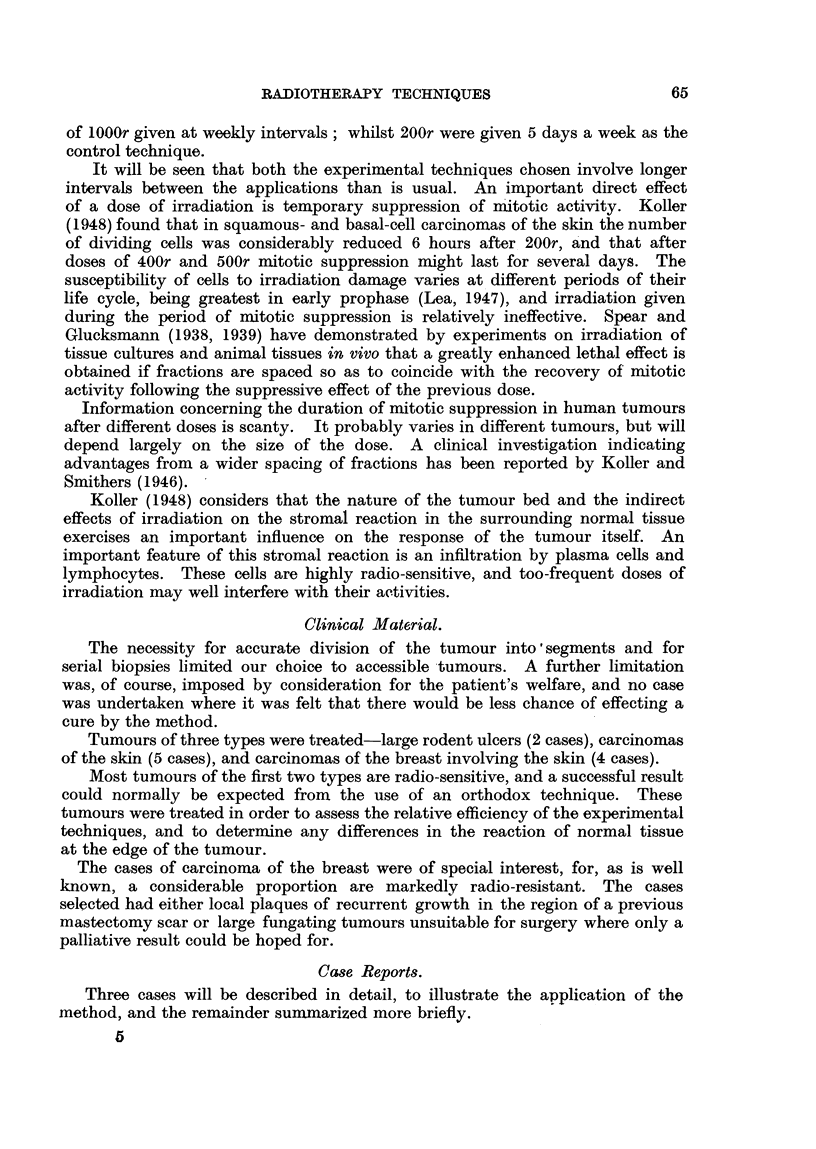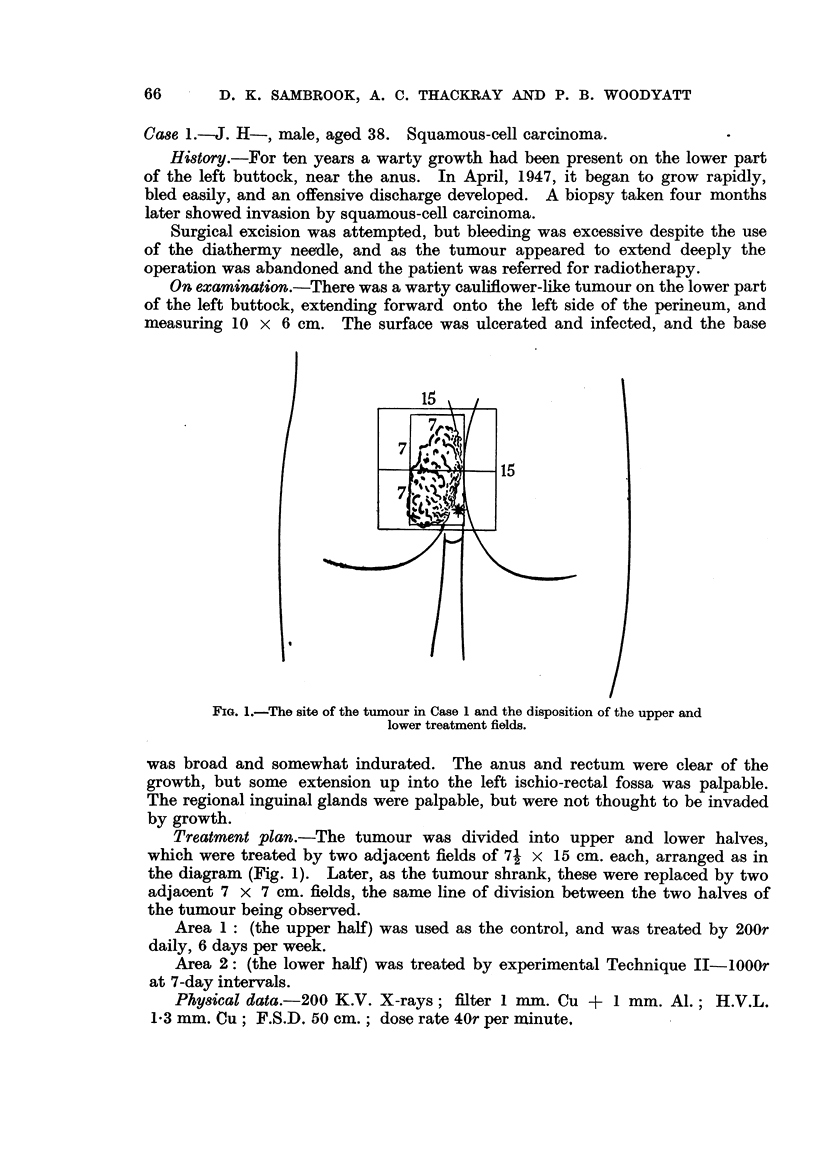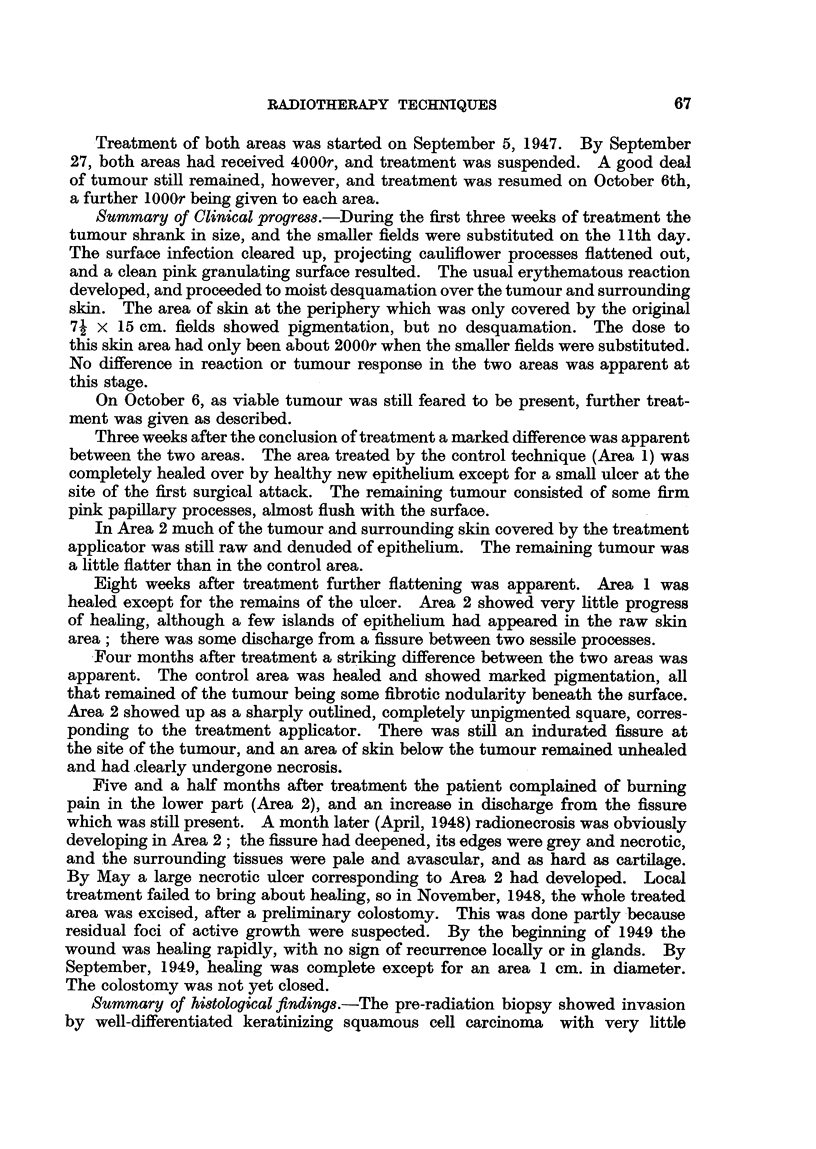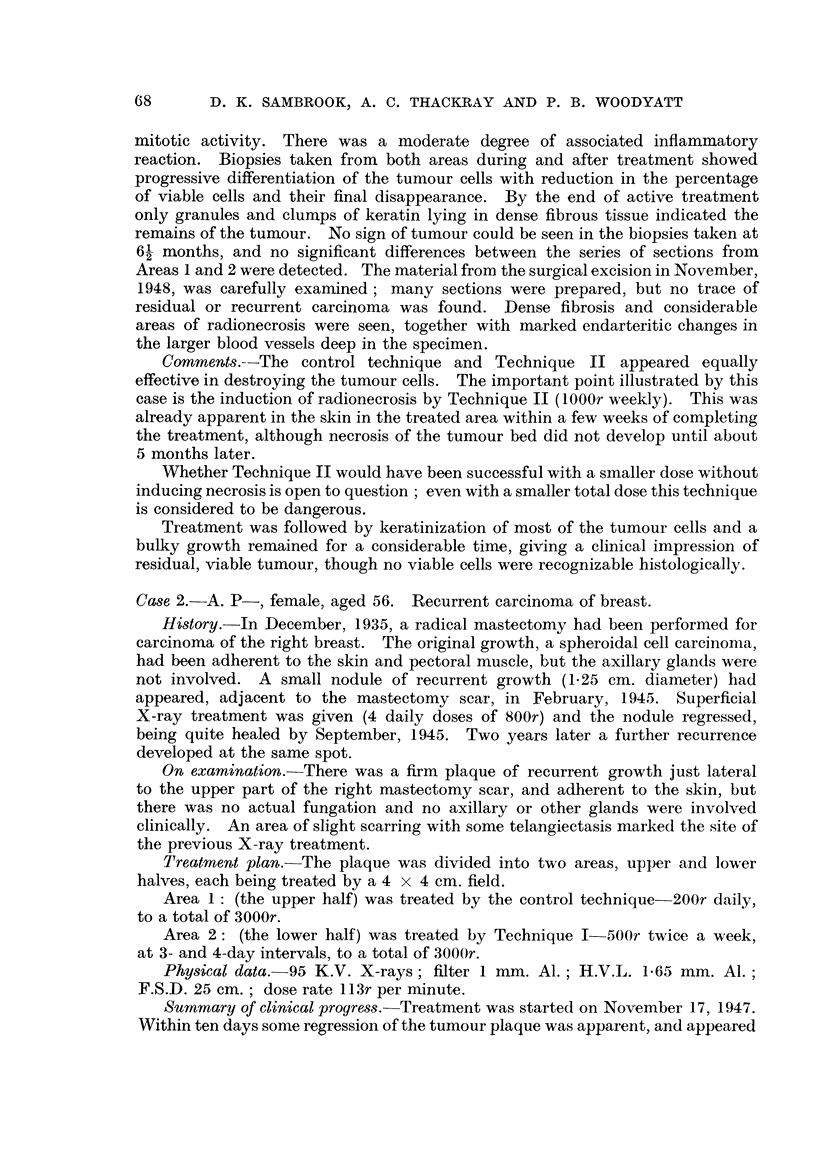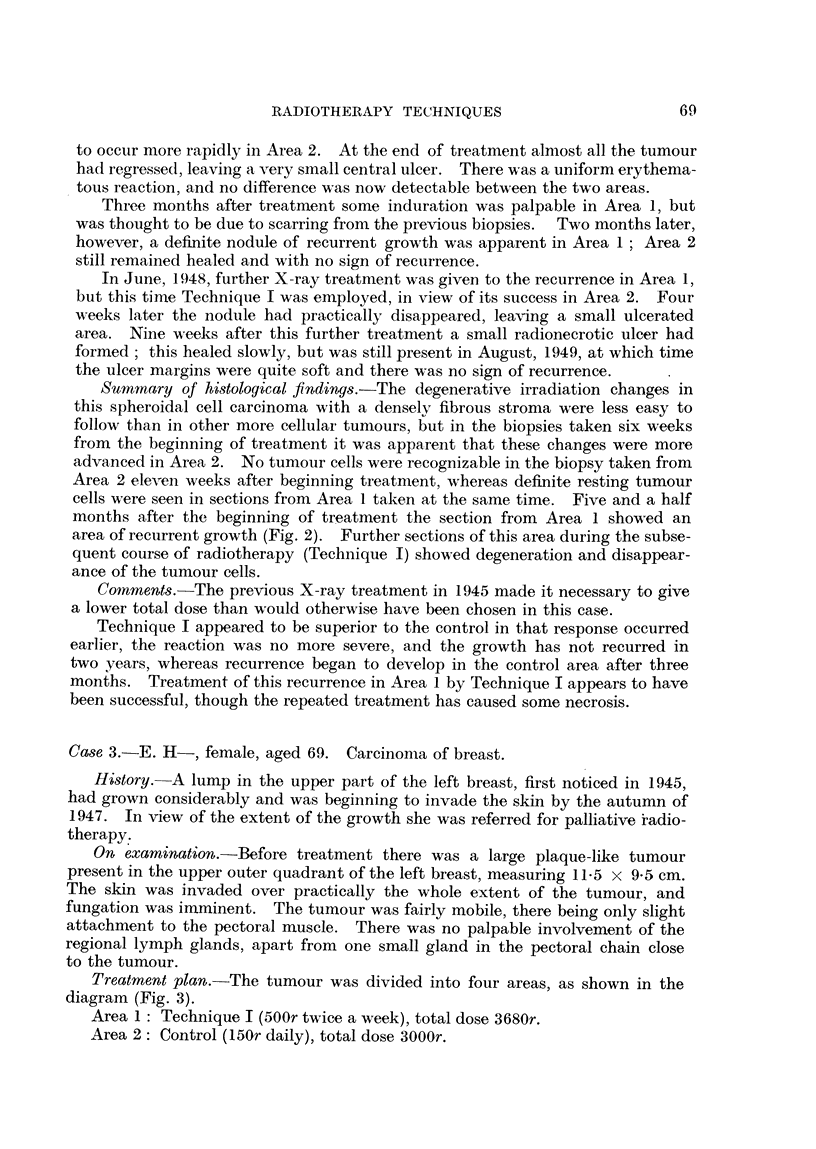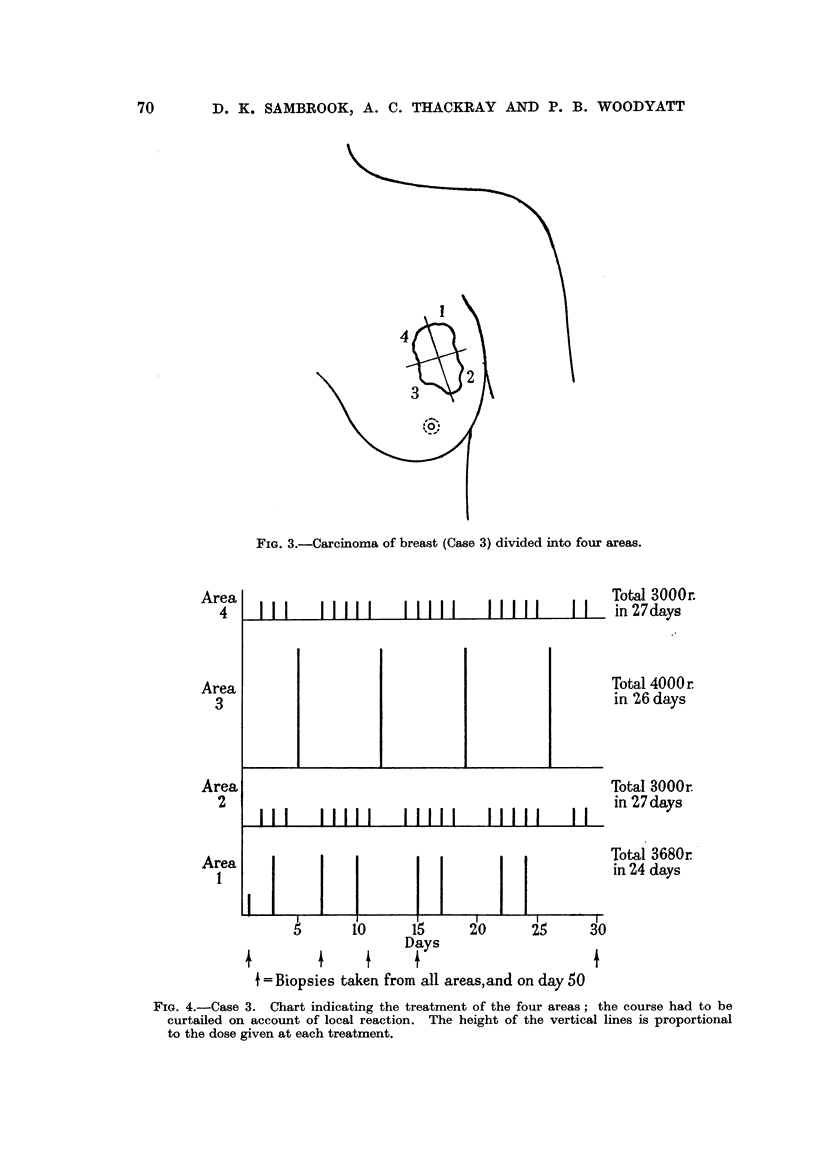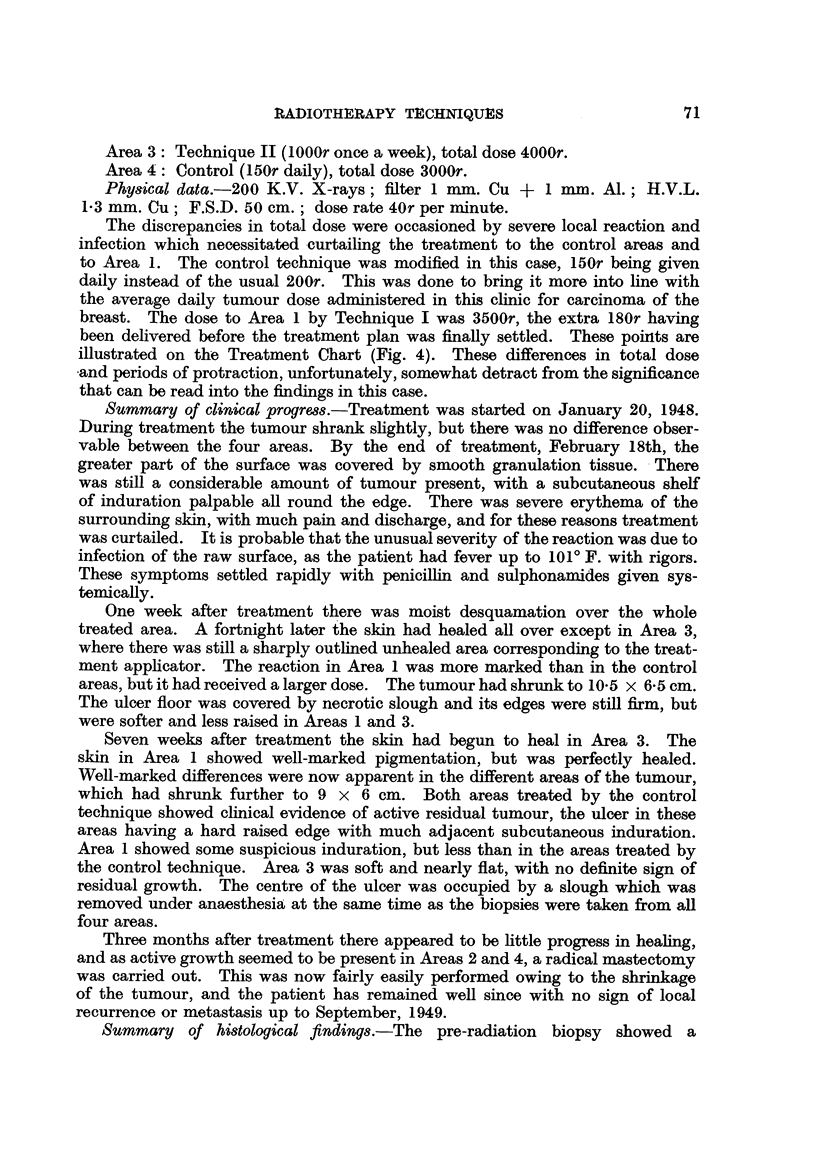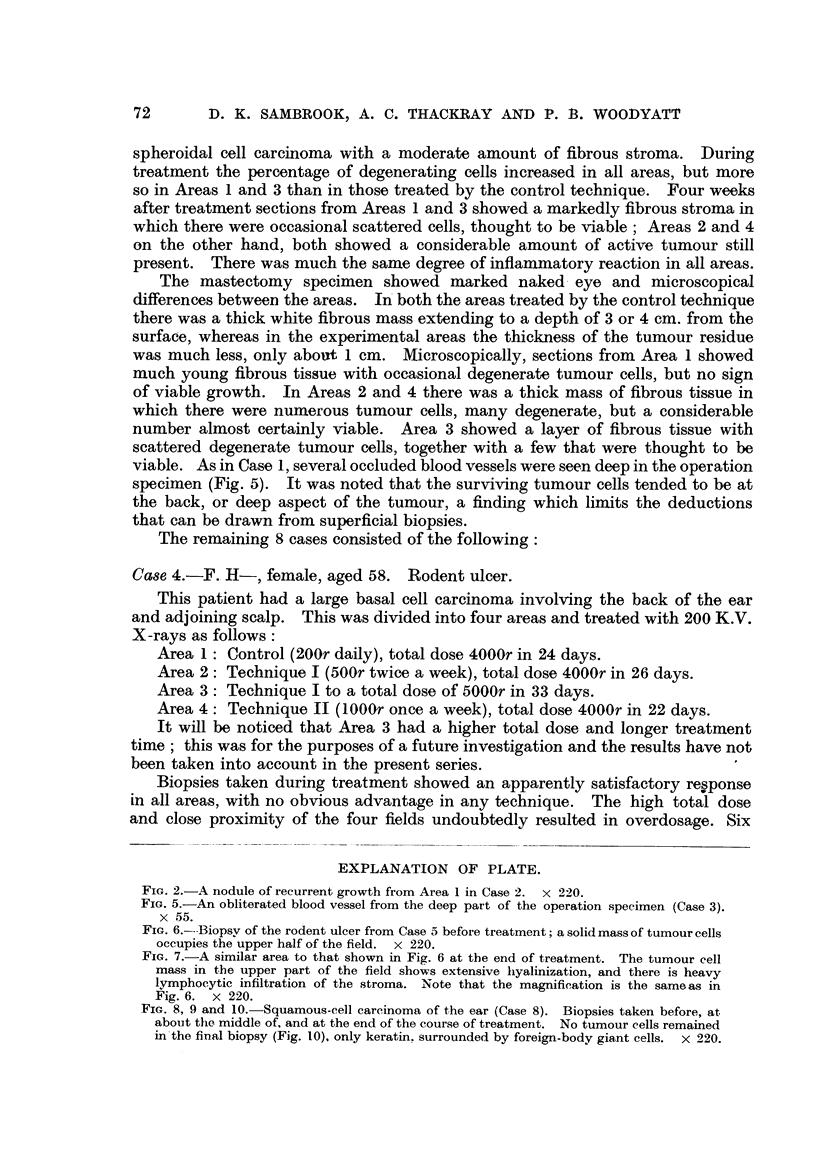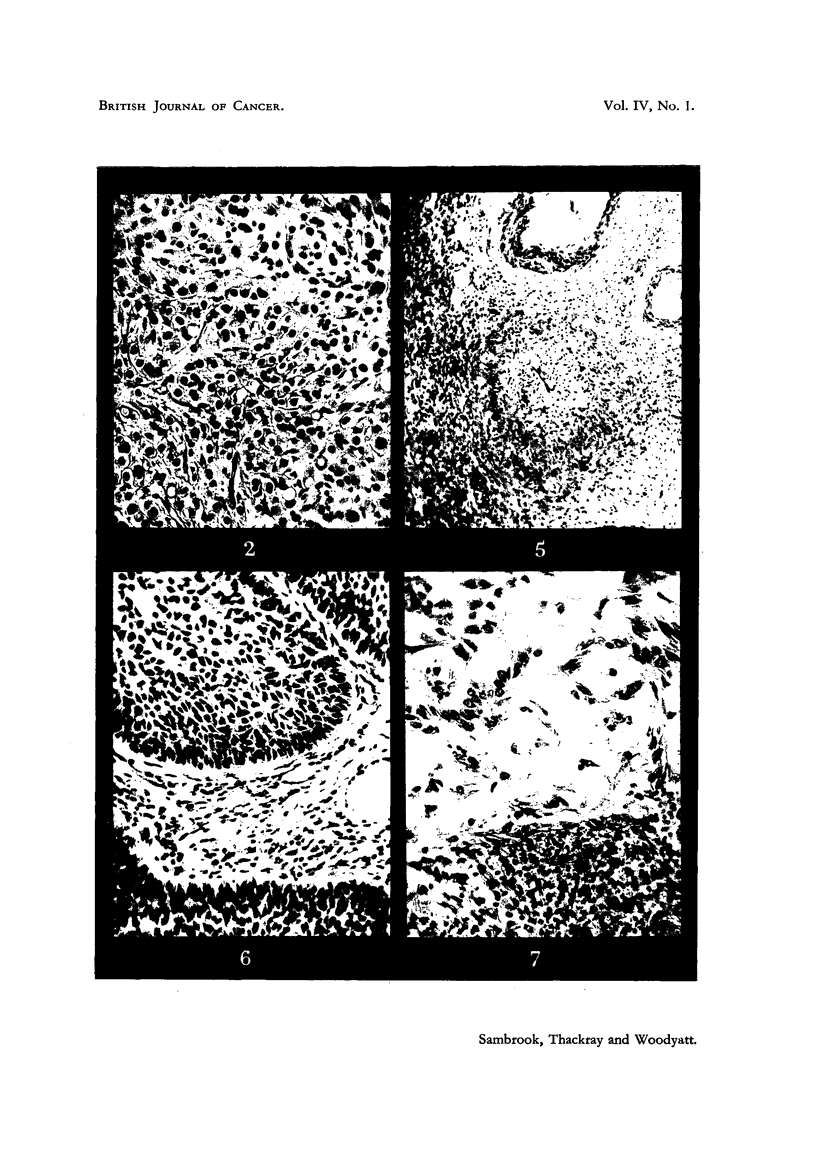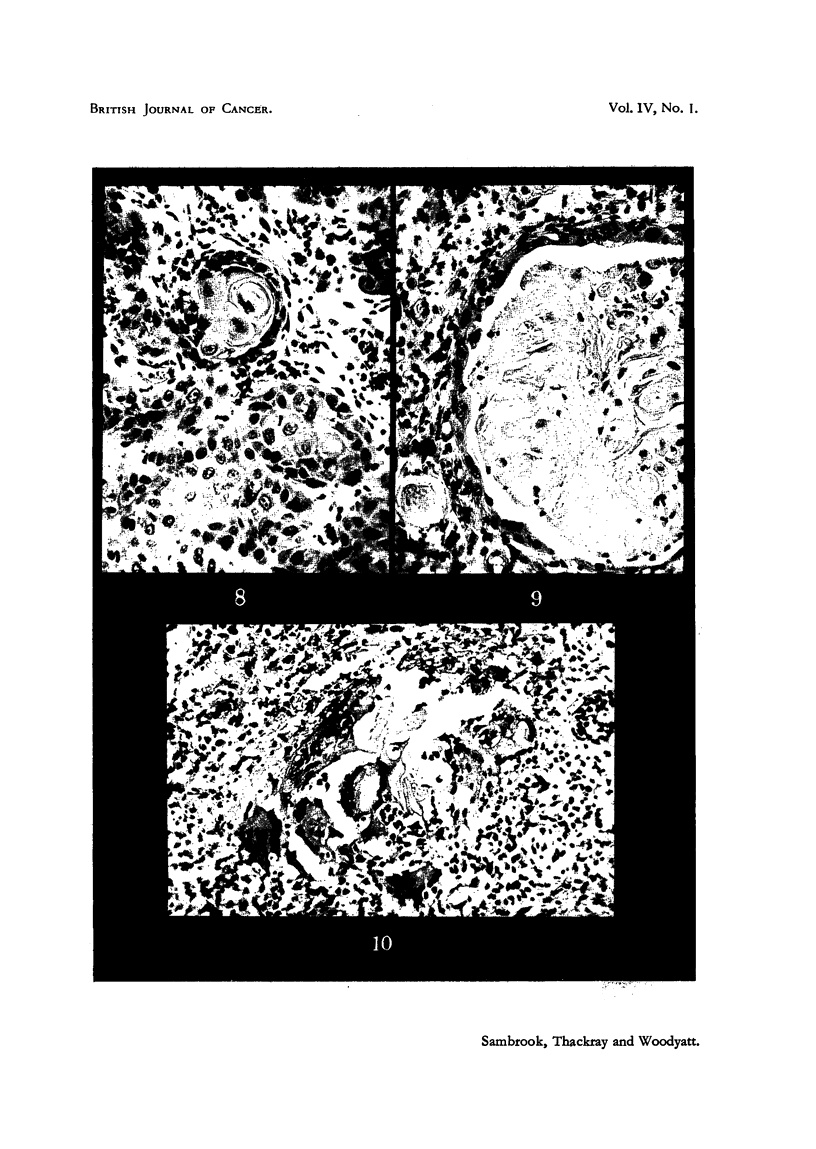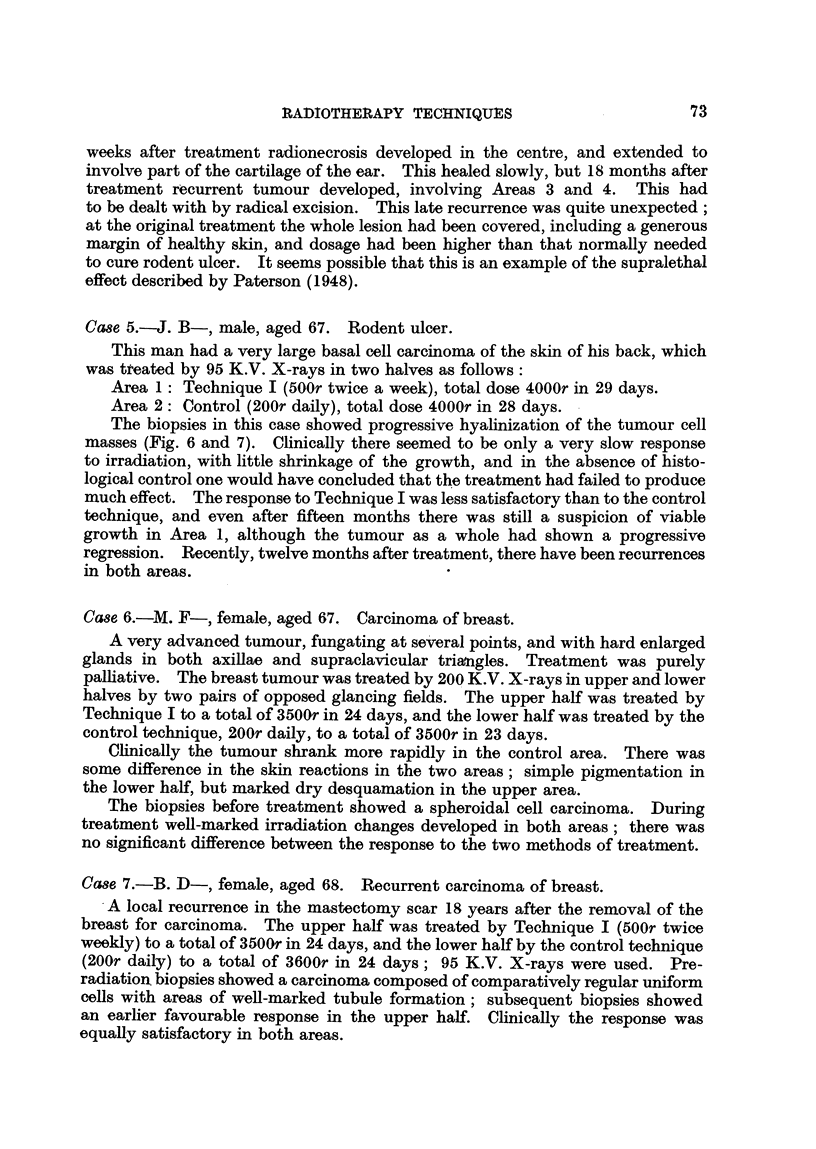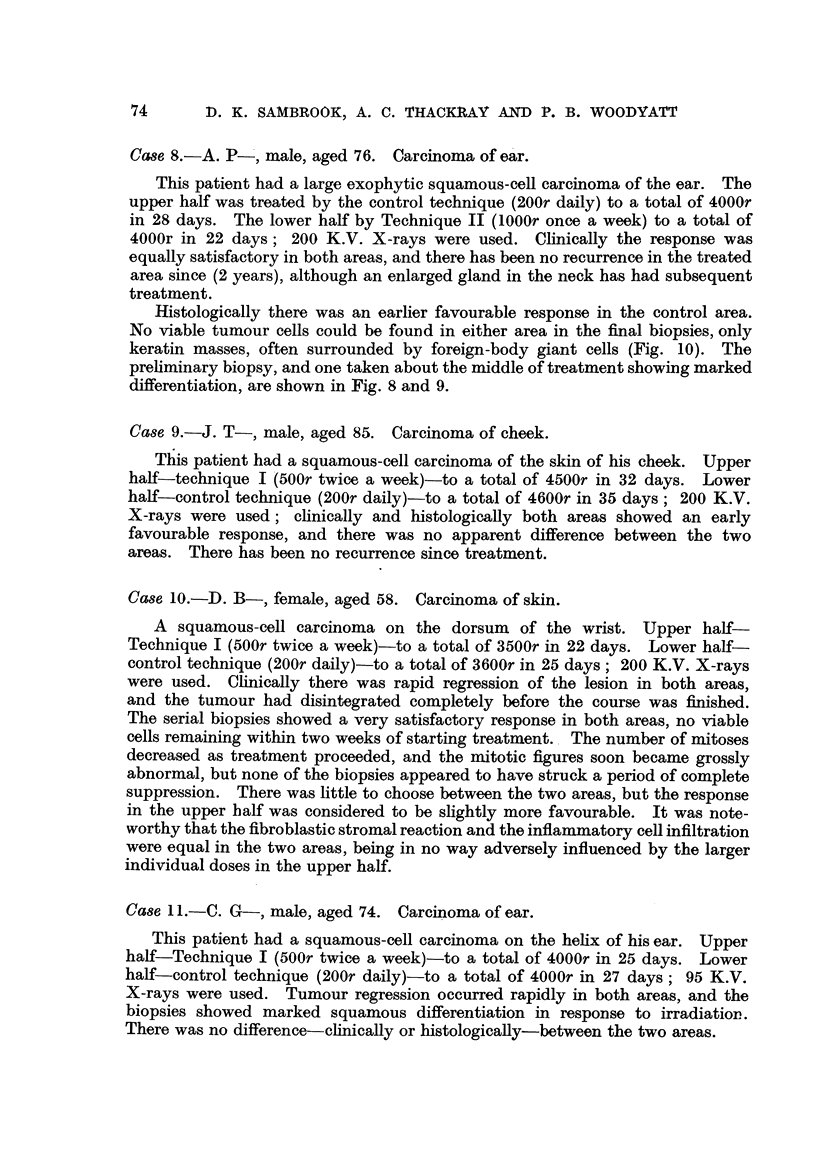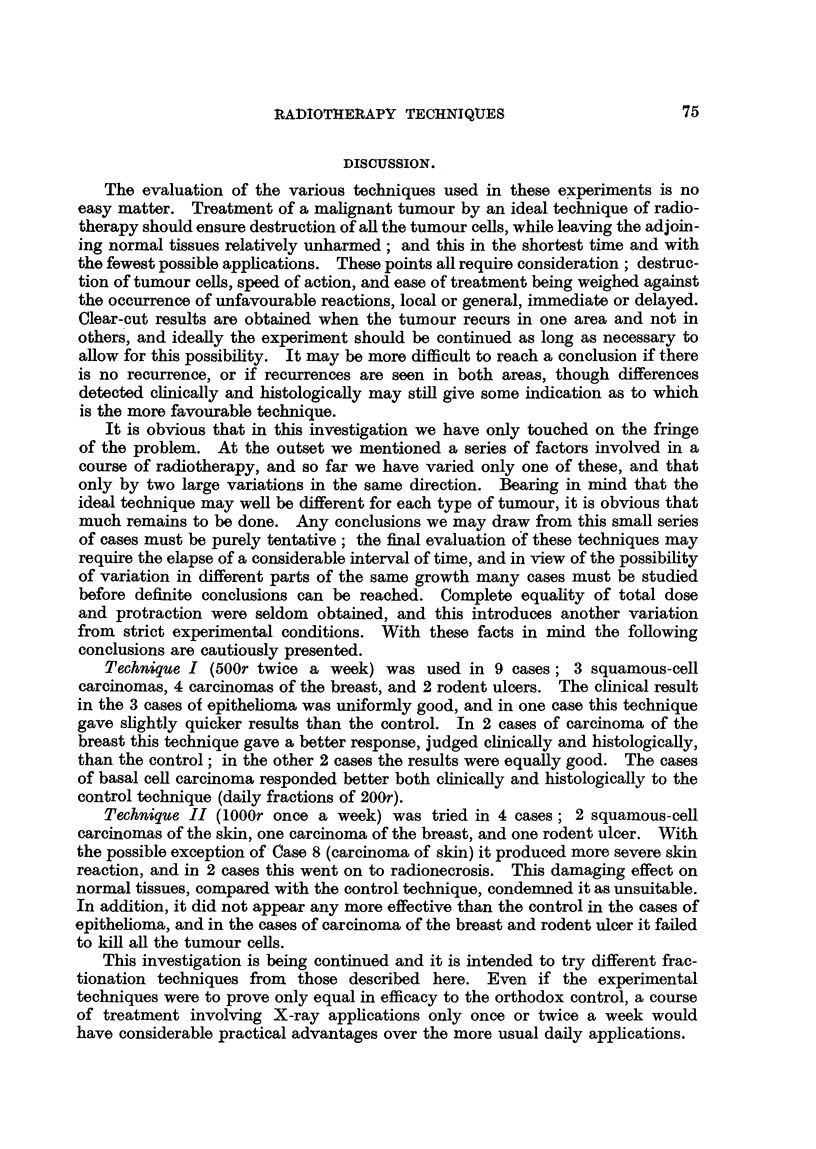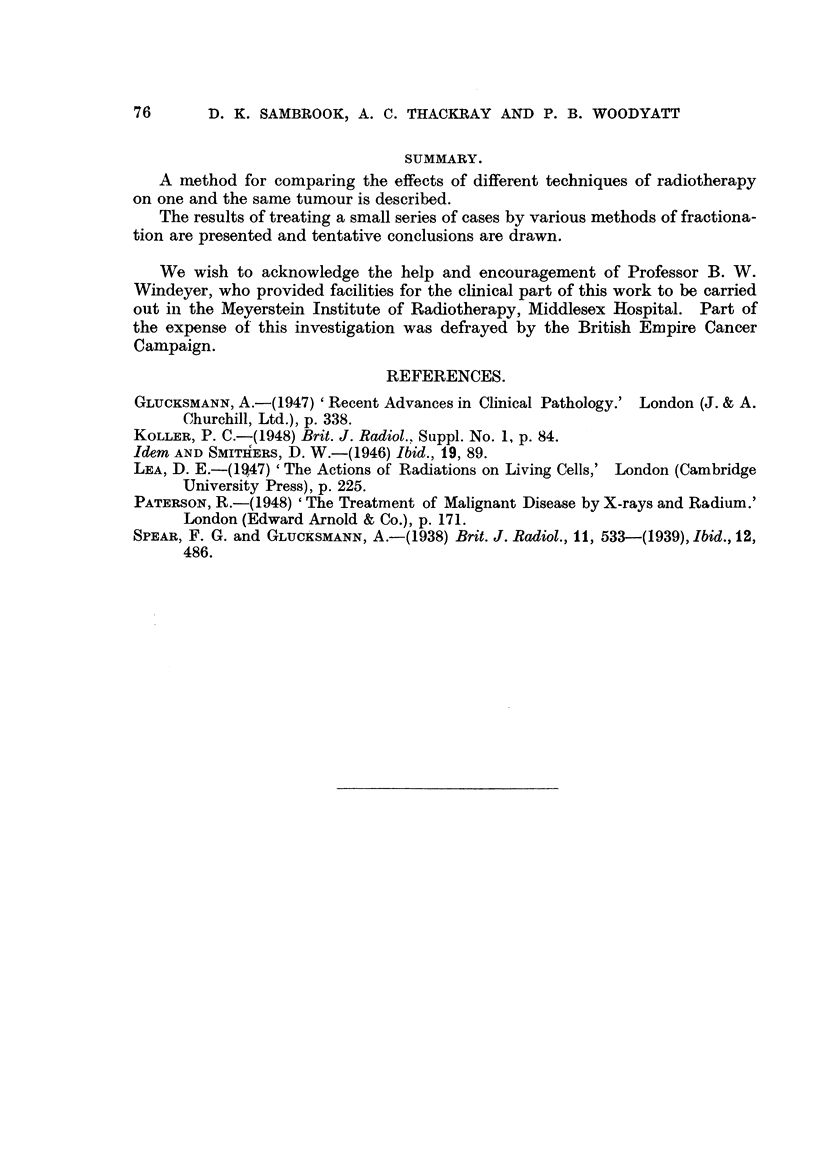# A Comparative Study of Radiotherapy Techniques

**DOI:** 10.1038/bjc.1950.5

**Published:** 1950-03

**Authors:** D. K. Sambrook, A. C. Thackray, P. B. Woodyatt

## Abstract

**Images:**


### A COMPARATIVE STUDY OF RADIOTHERAPY TECHNIQUES.

D. K. SAMBROOK,* A. C. THACKRAY, AND P. B. WOODYATT.*

*From the Meyerstein Institute of Radiotherapy and the Bland Sutton Institute of Pathology, Middlesex Hospital, London, W.1.*

Received for publication February 15, 1950.

FAILURE in the treatment of malignant disease is most often due to the occurrence of metastasis before treatment of the primary lesion is instituted. There are, however, a number of cases in which the tumour remains localized for a considerable time, but cannot be removed surgically on account of its size and anatomical situation, and cannot be destroyed by radiotherapy without irreparable damage to neighbouring normal tissues. It is the aim of the radiotherapist to exploit the differences in sensitivity between normal and neoplastic tissues, and it is the object of this research to see whether the effects of radiotherapy on certain tumours can be enhanced by modification of the techniques of therapy in common use at present.

A course of X-ray treatment involves a number of variable factors. Among these are the number and size of separate doses into which the treatment is divided (fractionation) ; the time spacing of individual fractions ; the total dose given ; the time over which it is spread (protraction) ; the intensity of irradiation during each dose, and the quality of the radiation used, depending on the voltage and filtration.

Clearly, it would be impossible to obtain intelligible results from varying more than one of these factors at a time, and in this investigation we have studied the effect of varying the fractionation.

This work is reported here, not so much for the results obtained, as to illustrate a method of investigation which may prove to be of value.

### METHOD.

Tumours, even of similar histological type, vary so much in their response to irradiation that it is of little value comparing the effects of different techniques of treatment on different tumours, even though these tumours appear to be very similar histologically.

It was therefore decided that comparisons of technique must be made on one and the same patient in each instance. For this purpose accessible tumours were selected that could be accurately divided into segments by appropriate skin markings. The segments, usually 2 or 4 in number, and as nearly as possible of equal size, were then treated by one or more experimental techniques, while one segment was treated by a control technique. Segments not being treated were protected by lead shields while neighbouring segments were irradiated, and care was taken to avoid overlap of adjacent segments or an untreated gap

* B.M.A. Research Scholars.

between them by careful skin markings and "setting up." The question of side-scattering of X-rays from one segment into adjacent ones was investigated by physical measurements with micro-ionization chambers in a wax "phantom." The amount of scattered radiation was found to be less than 5 per cent at 1 cm. from the edge of a treated segment, and this was considered small enough to be ignored.

In this way erroneous conclusions resulting from unpredictable individual variations in response are largely eliminated, and each case constitutes a complete separate experiment. The results of individual experiments can later be compared to see whether the experimental technique in question regularly produces a particular effect.

The tumours and surrounding skin were examined at frequent intervals to follow progress, and to detect and note differences in response between the areas. In addition, biopsies were taken from all areas before, during and after treatment. At first, weekly biopsies were taken, but it soon became obvious that in the earlier stages more frequent examinations were necessary, while after completion of the course of treatment intervals of a month or more might elapse. The sections were examined by a pathologist who not only had no preconceived views about the various techniques of irradiation employed, but was kept in ignorance of the area from which each specimen had been taken.

Biopsies from each area before treatment were studied carefully to provide a base line, and also to give an idea of the degree of variation between different areas of the same tumour. This variability was often found to be marked, and differences in biopsies subsequently taken from the several segments had often to be discounted as representing no more than possible chance variations. The changes usually described were seen in the biopsies as treatment proceeded, and the possibility of cell counts on the lines suggested by Glucksmann (1947) was considered to see if numerical comparisons of the efficacy of treatment could be made. However, the variation naturally occurring in different areas of the same growth, among other things, led us to abandon attempts at assigning figures to each biopsy as too difficult and time-consuming to carry out conscientiously and as liable to erroneous interpretation. Instead, a general impression of the changes, both in the tumour cells and in the stroma, was gained from the examination of each section, and only when favourable changes were regularly found to be more advanced in biopsies from any one area was the technique there employed regarded as superior on histological grounds.

### *Experimental Techniques Investigated.*

Conventional techniques of X-ray therapy of malignant tumours vary in detail in different centres, and according to the type of tumour treated. Fractionation is, however, usually by equal daily doses given on 5 or 6 days each week, and this fractionation technique is applied with little discrimination to widely different sorts of tumour.

Certain theoretical considerations, mentioned below, suggested that a different method of fractionation, leaving longer intervals between the fractions, might be more effective. Two experimental techniques were therefore chosen for trial; in each the protraction was at the rate of about 1,000*r* tumour dose per week, but the size of individual fractions and the intervals between them were varied. Technique I consisted of 500*r* given twice a week; Technique II consisted of 1000*r* given at weekly intervals ; whilst 200*r* were given 5 days a week as the control technique.

It will be seen that both the experimental techniques chosen involve longer intervals between the applications than is usual. An important direct effect of a dose of irradiation is temporary suppression of mitotic activity. Koller (1948) found that in squamous- and basal-cell carcinomas of the skin the number of dividing cells was considerably reduced 6 hours after 200*r*, and that after doses of 400*r* and 500*r* mitotic suppression might last for several days. The susceptibility of cells to irradiation damage varies at different periods of their life cycle, being greatest in early prophase (Lea, 1947), and irradiation given during the period of mitotic suppression is relatively ineffective. Spear and Glucksmann (1938, 1939) have demonstrated by experiments on irradiation of tissue cultures and animal tissues *in vivo* that a greatly enhanced lethal effect is obtained if fractions are spaced so as to coincide with the recovery of mitotic activity following the suppressive effect of the previous dose.

Information concerning the duration of mitotic suppression in human tumours after different doses is scanty. It probably varies in different tumours, but will depend largely on the size of the dose. A clinical investigation indicating advantages from a wider spacing of fractions has been reported by Koller and Smithers (1946).

Koller (1948) considers that the nature of the tumour bed and the indirect effects of irradiation on the stromal reaction in the surrounding normal tissue exercises an important influence on the response of the tumour itself. An important feature of this stromal reaction is an infiltration by plasma cells and lymphocytes. These cells are highly radio-sensitive, and too-frequent doses of irradiation may well interfere with their activities.

*Clinical Material.*

The necessity for accurate division of the tumour into segments and for serial biopsies limited our choice to accessible tumours. A further limitation was, of course, imposed by consideration for the patient's welfare, and no case was undertaken where it was felt that there would be less chance of effecting a cure by the method.

Tumours of three types were treated—large rodent ulcers (2 cases), carcinomas of the skin (5 cases), and carcinomas of the breast involving the skin (4 cases).

Most tumours of the first two types are radio-sensitive, and a successful result could normally be expected from the use of an orthodox technique. These tumours were treated in order to assess the relative efficiency of the experimental techniques, and to determine any differences in the reaction of normal tissue at the edge of the tumour.

The cases of carcinoma of the breast were of special interest, for, as is well known, a considerable proportion are markedly radio-resistant. The cases selected had either local plaques of recurrent growth in the region of a previous mastectomy scar or large fungating tumours unsuitable for surgery where only a palliative result could be hoped for.

*Case Reports.*

Three cases will be described in detail, to illustrate the application of the method, and the remainder summarized more briefly.

*Case* 1.—J. H—, male, aged 38. Squamous-cell carcinoma.

*History.*—For ten years a warty growth had been present on the lower part of the left buttock, near the anus. In April, 1947, it began to grow rapidly, bled easily, and an offensive discharge developed. A biopsy taken four months later showed invasion by squamous-cell carcinoma.

Surgical excision was attempted, but bleeding was excessive despite the use of the diathermy needle, and as the tumour appeared to extend deeply the operation was abandoned and the patient was referred for radiotherapy.

*On examination.*—There was a warty cauliflower-like tumour on the lower part of the left buttock, extending forward onto the left side of the perineum, and measuring 10 × 6 cm. The surface was ulcerated and infected, and the base

FIG. 1.—The site of the tumour in Case 1 and the disposition of the upper and lower treatment fields.

was broad and somewhat indurated. The anus and rectum were clear of the growth, but some extension up into the left ischio-rectal fossa was palpable. The regional inguinal glands were palpable, but were not thought to be invaded by growth.

*Treatment plan.*—The tumour was divided into upper and lower halves, which were treated by two adjacent fields of $7\frac{1}{2} \times 15$ cm. each, arranged as in the diagram (Fig. 1). Later, as the tumour shrank, these were replaced by two adjacent 7 × 7 cm. fields, the same line of division between the two halves of the tumour being observed.

Area 1 : (the upper half) was used as the control, and was treated by 200*r* daily, 6 days per week.

Area 2 : (the lower half) was treated by experimental Technique II—1000*r* at 7-day intervals.

*Physical data.*—200 K.V. X-rays ; filter 1 mm. Cu + 1 mm. Al. ; H.V.L. 1·3 mm. Cu ; F.S.D. 50 cm. ; dose rate 40*r* per minute.

Treatment of both areas was started on September 5, 1947. By September 27, both areas had received 4000*r*, and treatment was suspended. A good deal of tumour still remained, however, and treatment was resumed on October 6th, a further 1000*r* being given to each area.

*Summary of Clinical progress.*—During the first three weeks of treatment the tumour shrank in size, and the smaller fields were substituted on the 11th day. The surface infection cleared up, projecting cauliflower processes flattened out, and a clean pink granulating surface resulted. The usual erythematous reaction developed, and proceeded to moist desquamation over the tumour and surrounding skin. The area of skin at the periphery which was only covered by the original $7\frac{1}{2} \times 15$ cm. fields showed pigmentation, but no desquamation. The dose to this skin area had only been about 2000*r* when the smaller fields were substituted. No difference in reaction or tumour response in the two areas was apparent at this stage.

On October 6, as viable tumour was still feared to be present, further treatment was given as described.

Three weeks after the conclusion of treatment a marked difference was apparent between the two areas. The area treated by the control technique (Area 1) was completely healed over by healthy new epithelium except for a small ulcer at the site of the first surgical attack. The remaining tumour consisted of some firm pink papillary processes, almost flush with the surface.

In Area 2 much of the tumour and surrounding skin covered by the treatment applicator was still raw and denuded of epithelium. The remaining tumour was a little flatter than in the control area.

Eight weeks after treatment further flattening was apparent. Area 1 was healed except for the remains of the ulcer. Area 2 showed very little progress of healing, although a few islands of epithelium had appeared in the raw skin area ; there was some discharge from a fissure between two sessile processes.

Four months after treatment a striking difference between the two areas was apparent. The control area was healed and showed marked pigmentation, all that remained of the tumour being some fibrotic nodularity beneath the surface. Area 2 showed up as a sharply outlined, completely unpigmented square, corresponding to the treatment applicator. There was still an indurated fissure at the site of the tumour, and an area of skin below the tumour remained unhealed and had clearly undergone necrosis.

Five and a half months after treatment the patient complained of burning pain in the lower part (Area 2), and an increase in discharge from the fissure which was still present. A month later (April, 1948) radionecrosis was obviously developing in Area 2 ; the fissure had deepened, its edges were grey and necrotic, and the surrounding tissues were pale and avascular, and as hard as cartilage. By May a large necrotic ulcer corresponding to Area 2 had developed. Local treatment failed to bring about healing, so in November, 1948, the whole treated area was excised, after a preliminary colostomy. This was done partly because residual foci of active growth were suspected. By the beginning of 1949 the wound was healing rapidly, with no sign of recurrence locally or in glands. By September, 1949, healing was complete except for an area 1 cm. in diameter. The colostomy was not yet closed.

*Summary of histological findings.*—The pre-radiation biopsy showed invasion by well-differentiated keratinizing squamous cell carcinoma with very little mitotic activity. There was a moderate degree of associated inflammatory reaction. Biopsies taken from both areas during and after treatment showed progressive differentiation of the tumour cells with reduction in the percentage of viable cells and their final disappearance. By the end of active treatment only granules and clumps of keratin lying in dense fibrous tissue indicated the remains of the tumour. No sign of tumour could be seen in the biopsies taken at $6\frac{1}{2}$ months, and no significant differences between the series of sections from Areas 1 and 2 were detected. The material from the surgical excision in November, 1948, was carefully examined ; many sections were prepared, but no trace of residual or recurrent carcinoma was found. Dense fibrosis and considerable areas of radionecrosis were seen, together with marked endarteritic changes in the larger blood vessels deep in the specimen.

*Comments.*—The control technique and Technique II appeared equally effective in destroying the tumour cells. The important point illustrated by this case is the induction of radionecrosis by Technique II (1000*r* weekly). This was already apparent in the skin in the treated area within a few weeks of completing the treatment, although necrosis of the tumour bed did not develop until about 5 months later.

Whether Technique II would have been successful with a smaller dose without inducing necrosis is open to question ; even with a smaller total dose this technique is considered to be dangerous.

Treatment was followed by keratinization of most of the tumour cells and a bulky growth remained for a considerable time, giving a clinical impression of residual, viable tumour, though no viable cells were recognizable histologically.

*Case* 2.—A. P—, female, aged 56. Recurrent carcinoma of breast.

*History.*—In December, 1935, a radical mastectomy had been performed for carcinoma of the right breast. The original growth, a spheroidal cell carcinoma, had been adherent to the skin and pectoral muscle, but the axillary glands were not involved. A small nodule of recurrent growth (1·25 cm. diameter) had appeared, adjacent to the mastectomy scar, in February, 1945. Superficial X-ray treatment was given (4 daily doses of 800*r*) and the nodule regressed, being quite healed by September, 1945. Two years later a further recurrence developed at the same spot.

*On examination.*—There was a firm plaque of recurrent growth just lateral to the upper part of the right mastectomy scar, and adherent to the skin, but there was no actual fungation and no axillary or other glands were involved clinically. An area of slight scarring with some telangiectasis marked the site of the previous X-ray treatment.

*Treatment plan.*—The plaque was divided into two areas, upper and lower halves, each being treated by a 4 $\times$ 4 cm. field.

Area 1 : (the upper half) was treated by the control technique—200*r* daily, to a total of 3000*r*.

Area 2 : (the lower half) was treated by Technique I—500*r* twice a week, at 3- and 4-day intervals, to a total of 3000*r*.

*Physical data.*—95 K.V. X-rays ; filter 1 mm. Al. ; H.V.L. 1·65 mm. Al. ; F.S.D. 25 cm. ; dose rate 113*r* per minute.

*Summary of clinical progress.*—Treatment was started on November 17, 1947. Within ten days some regression of the tumour plaque was apparent, and appeared to occur more rapidly in Area 2. At the end of treatment almost all the tumour had regressed, leaving a very small central ulcer. There was a uniform erythematous reaction, and no difference was now detectable between the two areas.

Three months after treatment some induration was palpable in Area 1, but was thought to be due to scarring from the previous biopsies. Two months later, however, a definite nodule of recurrent growth was apparent in Area 1 ; Area 2 still remained healed and with no sign of recurrence.

In June, 1948, further X-ray treatment was given to the recurrence in Area 1, but this time Technique I was employed, in view of its success in Area 2. Four weeks later the nodule had practically disappeared, leaving a small ulcerated area. Nine weeks after this further treatment a small radionecrotic ulcer had formed ; this healed slowly, but was still present in August, 1949, at which time the ulcer margins were quite soft and there was no sign of recurrence.

*Summary of histological findings.*—The degenerative irradiation changes in this spheroidal cell carcinoma with a densely fibrous stroma were less easy to follow than in other more cellular tumours, but in the biopsies taken six weeks from the beginning of treatment it was apparent that these changes were more advanced in Area 2. No tumour cells were recognizable in the biopsy taken from Area 2 eleven weeks after beginning treatment, whereas definite resting tumour cells were seen in sections from Area 1 taken at the same time. Five and a half months after the beginning of treatment the section from Area 1 showed an area of recurrent growth (Fig. 2). Further sections of this area during the subsequent course of radiotherapy (Technique I) showed degeneration and disappearance of the tumour cells.

*Comments.*—The previous X-ray treatment in 1945 made it necessary to give a lower total dose than would otherwise have been chosen in this case.

Technique I appeared to be superior to the control in that response occurred earlier, the reaction was no more severe, and the growth has not recurred in two years, whereas recurrence began to develop in the control area after three months. Treatment of this recurrence in Area 1 by Technique I appears to have been successful, though the repeated treatment has caused some necrosis.

*Case* 3.—E. H—, female, aged 69. Carcinoma of breast.

*History.*—A lump in the upper part of the left breast, first noticed in 1945, had grown considerably and was beginning to invade the skin by the autumn of 1947. In view of the extent of the growth she was referred for palliative radiotherapy.

*On examination.*—Before treatment there was a large plaque-like tumour present in the upper outer quadrant of the left breast, measuring 11·5 × 9·5 cm. The skin was invaded over practically the whole extent of the tumour, and fungation was imminent. The tumour was fairly mobile, there being only slight attachment to the pectoral muscle. There was no palpable involvement of the regional lymph glands, apart from one small gland in the pectoral chain close to the tumour.

*Treatment plan.*—The tumour was divided into four areas, as shown in the diagram (Fig. 3).

Area 1 : Technique I (500*r* twice a week), total dose 3680*r*.

Area 2 : Control (150*r* daily), total dose 3000*r*.

FIG. 3.—Carcinoma of breast (Case 3) divided into four areas.

FIG. 4.—Case 3. Chart indicating the treatment of the four areas ; the course had to be curtailed on account of local reaction. The height of the vertical lines is proportional to the dose given at each treatment.

Area 3 : Technique II (1000r once a week), total dose 4000r.

Area 4 : Control (150r daily), total dose 3000r.

*Physical data.*—200 K.V. X-rays ; filter 1 mm. Cu + 1 mm. Al. ; H.V.L. 1·3 mm. Cu ; F.S.D. 50 cm. ; dose rate 40r per minute.

The discrepancies in total dose were occasioned by severe local reaction and infection which necessitated curtailing the treatment to the control areas and to Area 1. The control technique was modified in this case, 150r being given daily instead of the usual 200r. This was done to bring it more into line with the average daily tumour dose administered in this clinic for carcinoma of the breast. The dose to Area 1 by Technique I was 3500r, the extra 180r having been delivered before the treatment plan was finally settled. These points are illustrated on the Treatment Chart (Fig. 4). These differences in total dose and periods of protraction, unfortunately, somewhat detract from the significance that can be read into the findings in this case.

*Summary of clinical progress.*—Treatment was started on January 20, 1948. During treatment the tumour shrank slightly, but there was no difference observable between the four areas. By the end of treatment, February 18th, the greater part of the surface was covered by smooth granulation tissue. There was still a considerable amount of tumour present, with a subcutaneous shelf of induration palpable all round the edge. There was severe erythema of the surrounding skin, with much pain and discharge, and for these reasons treatment was curtailed. It is probable that the unusual severity of the reaction was due to infection of the raw surface, as the patient had fever up to 101° F. with rigors. These symptoms settled rapidly with penicillin and sulphonamides given systemically.

One week after treatment there was moist desquamation over the whole treated area. A fortnight later the skin had healed all over except in Area 3, where there was still a sharply outlined unhealed area corresponding to the treatment applicator. The reaction in Area 1 was more marked than in the control areas, but it had received a larger dose. The tumour had shrunk to 10·5 × 6·5 cm. The ulcer floor was covered by necrotic slough and its edges were still firm, but were softer and less raised in Areas 1 and 3.

Seven weeks after treatment the skin had begun to heal in Area 3. The skin in Area 1 showed well-marked pigmentation, but was perfectly healed. Well-marked differences were now apparent in the different areas of the tumour, which had shrunk further to 9 × 6 cm. Both areas treated by the control technique showed clinical evidence of active residual tumour, the ulcer in these areas having a hard raised edge with much adjacent subcutaneous induration. Area 1 showed some suspicious induration, but less than in the areas treated by the control technique. Area 3 was soft and nearly flat, with no definite sign of residual growth. The centre of the ulcer was occupied by a slough which was removed under anaesthesia at the same time as the biopsies were taken from all four areas.

Three months after treatment there appeared to be little progress in healing, and as active growth seemed to be present in Areas 2 and 4, a radical mastectomy was carried out. This was now fairly easily performed owing to the shrinkage of the tumour, and the patient has remained well since with no sign of local recurrence or metastasis up to September, 1949.

*Summary of histological findings.*—The pre-radiation biopsy showed a spheroidal cell carcinoma with a moderate amount of fibrous stroma. During treatment the percentage of degenerating cells increased in all areas, but more so in Areas 1 and 3 than in those treated by the control technique. Four weeks after treatment sections from Areas 1 and 3 showed a markedly fibrous stroma in which there were occasional scattered cells, thought to be viable ; Areas 2 and 4 on the other hand, both showed a considerable amount of active tumour still present. There was much the same degree of inflammatory reaction in all areas.

The mastectomy specimen showed marked naked eye and microscopical differences between the areas. In both the areas treated by the control technique there was a thick white fibrous mass extending to a depth of 3 or 4 cm. from the surface, whereas in the experimental areas the thickness of the tumour residue was much less, only about 1 cm. Microscopically, sections from Area 1 showed much young fibrous tissue with occasional degenerate tumour cells, but no sign of viable growth. In Areas 2 and 4 there was a thick mass of fibrous tissue in which there were numerous tumour cells, many degenerate, but a considerable number almost certainly viable. Area 3 showed a layer of fibrous tissue with scattered degenerate tumour cells, together with a few that were thought to be viable. As in Case 1, several occluded blood vessels were seen deep in the operation specimen (Fig. 5). It was noted that the surviving tumour cells tended to be at the back, or deep aspect of the tumour, a finding which limits the deductions that can be drawn from superficial biopsies.

The remaining 8 cases consisted of the following :

*Case* 4.—F. H—, female, aged 58. Rodent ulcer.

This patient had a large basal cell carcinoma involving the back of the ear and adjoining scalp. This was divided into four areas and treated with 200 K.V. X-rays as follows :

Area 1 : Control (200*r* daily), total dose 4000*r* in 24 days.
Area 2 : Technique I (500*r* twice a week), total dose 4000*r* in 26 days.
Area 3 : Technique I to a total dose of 5000*r* in 33 days.
Area 4 : Technique II (1000*r* once a week), total dose 4000*r* in 22 days.

It will be noticed that Area 3 had a higher total dose and longer treatment time ; this was for the purposes of a future investigation and the results have not been taken into account in the present series.

Biopsies taken during treatment showed an apparently satisfactory response in all areas, with no obvious advantage in any technique. The high total dose and close proximity of the four fields undoubtedly resulted in overdosage. Six

---

EXPLANATION OF PLATE.

Fig. 2.—A nodule of recurrent growth from Area 1 in Case 2. × 220.

Fig. 5.—An obliterated blood vessel from the deep part of the operation specimen (Case 3). × 55.

Fig. 6.—Biopsy of the rodent ulcer from Case 5 before treatment; a solid mass of tumour cells occupies the upper half of the field. × 220.

Fig. 7.—A similar area to that shown in Fig. 6 at the end of treatment. The tumour cell mass in the upper part of the field shows extensive hyalinization, and there is heavy lymphocytic infiltration of the stroma. Note that the magnification is the same as in Fig. 6. × 220.

Fig. 8, 9 and 10.—Squamous-cell carcinoma of the ear (Case 8). Biopsies taken before, at about the middle of, and at the end of the course of treatment. No tumour cells remained in the final biopsy (Fig. 10), only keratin, surrounded by foreign-body giant cells. × 220.

Sambrook, Thackray and Woodyatt.

8

9

10

Sambrook, Thackray and Woodyatt.

weeks after treatment radionecrosis developed in the centre, and extended to involve part of the cartilage of the ear. This healed slowly, but 18 months after treatment recurrent tumour developed, involving Areas 3 and 4. This had to be dealt with by radical excision. This late recurrence was quite unexpected ; at the original treatment the whole lesion had been covered, including a generous margin of healthy skin, and dosage had been higher than that normally needed to cure rodent ulcer. It seems possible that this is an example of the supralethal effect described by Paterson (1948).

*Case* 5.—J. B—, male, aged 67. Rodent ulcer.

This man had a very large basal cell carcinoma of the skin of his back, which was treated by 95 K.V. X-rays in two halves as follows :

Area 1 : Technique I (500*r* twice a week), total dose 4000*r* in 29 days.

Area 2 : Control (200*r* daily), total dose 4000*r* in 28 days.

The biopsies in this case showed progressive hyalinization of the tumour cell masses (Fig. 6 and 7). Clinically there seemed to be only a very slow response to irradiation, with little shrinkage of the growth, and in the absence of histological control one would have concluded that the treatment had failed to produce much effect. The response to Technique I was less satisfactory than to the control technique, and even after fifteen months there was still a suspicion of viable growth in Area 1, although the tumour as a whole had shown a progressive regression. Recently, twelve months after treatment, there have been recurrences in both areas.

*Case* 6.—M. F—, female, aged 67. Carcinoma of breast.

A very advanced tumour, fungating at several points, and with hard enlarged glands in both axillae and supraclavicular triangles. Treatment was purely palliative. The breast tumour was treated by 200 K.V. X-rays in upper and lower halves by two pairs of opposed glancing fields. The upper half was treated by Technique I to a total of 3500*r* in 24 days, and the lower half was treated by the control technique, 200*r* daily, to a total of 3500*r* in 23 days.

Clinically the tumour shrank more rapidly in the control area. There was some difference in the skin reactions in the two areas ; simple pigmentation in the lower half, but marked dry desquamation in the upper area.

The biopsies before treatment showed a spheroidal cell carcinoma. During treatment well-marked irradiation changes developed in both areas ; there was no significant difference between the response to the two methods of treatment.

*Case* 7.—B. D—, female, aged 68. Recurrent carcinoma of breast.

A local recurrence in the mastectomy scar 18 years after the removal of the breast for carcinoma. The upper half was treated by Technique I (500*r* twice weekly) to a total of 3500*r* in 24 days, and the lower half by the control technique (200*r* daily) to a total of 3600*r* in 24 days ; 95 K.V. X-rays were used. Preradiation biopsies showed a carcinoma composed of comparatively regular uniform cells with areas of well-marked tubule formation ; subsequent biopsies showed an earlier favourable response in the upper half. Clinically the response was equally satisfactory in both areas.

*Case* 8.—A. P—, male, aged 76. Carcinoma of ear.

This patient had a large exophytic squamous-cell carcinoma of the ear. The upper half was treated by the control technique (200*r* daily) to a total of 4000*r* in 28 days. The lower half by Technique II (1000*r* once a week) to a total of 4000r in 22 days; 200 K.V. X-rays were used. Clinically the response was equally satisfactory in both areas, and there has been no recurrence in the treated area since (2 years), although an enlarged gland in the neck has had subsequent treatment.

Histologically there was an earlier favourable response in the control area. No viable tumour cells could be found in either area in the final biopsies, only keratin masses, often surrounded by foreign-body giant cells (Fig. 10). The preliminary biopsy, and one taken about the middle of treatment showing marked differentiation, are shown in Fig. 8 and 9.

*Case* 9.—J. T—, male, aged 85. Carcinoma of cheek.

This patient had a squamous-cell carcinoma of the skin of his cheek. Upper half—technique I (500*r* twice a week)—to a total of 4500*r* in 32 days. Lower half—control technique (200*r* daily)—to a total of 4600*r* in 35 days; 200 K.V. X-rays were used; clinically and histologically both areas showed an early favourable response, and there was no apparent difference between the two areas. There has been no recurrence since treatment.

*Case* 10.—D. B—, female, aged 58. Carcinoma of skin.

A squamous-cell carcinoma on the dorsum of the wrist. Upper half—Technique I (500*r* twice a week)—to a total of 3500*r* in 22 days. Lower half—control technique (200*r* daily)—to a total of 3600*r* in 25 days; 200 K.V. X-rays were used. Clinically there was rapid regression of the lesion in both areas, and the tumour had disintegrated completely before the course was finished. The serial biopsies showed a very satisfactory response in both areas, no viable cells remaining within two weeks of starting treatment. The number of mitoses decreased as treatment proceeded, and the mitotic figures soon became grossly abnormal, but none of the biopsies appeared to have struck a period of complete suppression. There was little to choose between the two areas, but the response in the upper half was considered to be slightly more favourable. It was noteworthy that the fibroblastic stromal reaction and the inflammatory cell infiltration were equal in the two areas, being in no way adversely influenced by the larger individual doses in the upper half.

*Case* 11.—C. G—, male, aged 74. Carcinoma of ear.

This patient had a squamous-cell carcinoma on the helix of his ear. Upper half—Technique I (500*r* twice a week)—to a total of 4000*r* in 25 days. Lower half—control technique (200*r* daily)—to a total of 4000*r* in 27 days; 95 K.V. X-rays were used. Tumour regression occurred rapidly in both areas, and the biopsies showed marked squamous differentiation in response to irradiation. There was no difference—clinically or histologically—between the two areas.

### DISCUSSION.

The evaluation of the various techniques used in these experiments is no easy matter. Treatment of a malignant tumour by an ideal technique of radiotherapy should ensure destruction of all the tumour cells, while leaving the adjoining normal tissues relatively unharmed ; and this in the shortest time and with the fewest possible applications. These points all require consideration ; destruction of tumour cells, speed of action, and ease of treatment being weighed against the occurrence of unfavourable reactions, local or general, immediate or delayed. Clear-cut results are obtained when the tumour recurs in one area and not in others, and ideally the experiment should be continued as long as necessary to allow for this possibility. It may be more difficult to reach a conclusion if there is no recurrence, or if recurrences are seen in both areas, though differences detected clinically and histologically may still give some indication as to which is the more favourable technique.

It is obvious that in this investigation we have only touched on the fringe of the problem. At the outset we mentioned a series of factors involved in a course of radiotherapy, and so far we have varied only one of these, and that only by two large variations in the same direction. Bearing in mind that the ideal technique may well be different for each type of tumour, it is obvious that much remains to be done. Any conclusions we may draw from this small series of cases must be purely tentative ; the final evaluation of these techniques may require the elapse of a considerable interval of time, and in view of the possibility of variation in different parts of the same growth many cases must be studied before definite conclusions can be reached. Complete equality of total dose and protraction were seldom obtained, and this introduces another variation from strict experimental conditions. With these facts in mind the following conclusions are cautiously presented.

*Technique I* (500*r* twice a week) was used in 9 cases ; 3 squamous-cell carcinomas, 4 carcinomas of the breast, and 2 rodent ulcers. The clinical result in the 3 cases of epithelioma was uniformly good, and in one case this technique gave slightly quicker results than the control. In 2 cases of carcinoma of the breast this technique gave a better response, judged clinically and histologically, than the control ; in the other 2 cases the results were equally good. The cases of basal cell carcinoma responded better both clinically and histologically to the control technique (daily fractions of 200*r*).

*Technique II* (1000*r* once a week) was tried in 4 cases ; 2 squamous-cell carcinomas of the skin, one carcinoma of the breast, and one rodent ulcer. With the possible exception of Case 8 (carcinoma of skin) it produced more severe skin reaction, and in 2 cases this went on to radionecrosis. This damaging effect on normal tissues, compared with the control technique, condemned it as unsuitable. In addition, it did not appear any more effective than the control in the cases of epithelioma, and in the cases of carcinoma of the breast and rodent ulcer it failed to kill all the tumour cells.

This investigation is being continued and it is intended to try different fractionation techniques from those described here. Even if the experimental techniques were to prove only equal in efficacy to the orthodox control, a course of treatment involving X-ray applications only once or twice a week would have considerable practical advantages over the more usual daily applications.

SUMMARY.

A method for comparing the effects of different techniques of radiotherapy on one and the same tumour is described.

The results of treating a small series of cases by various methods of fractionation are presented and tentative conclusions are drawn.

We wish to acknowledge the help and encouragement of Professor B. W. Windeyer, who provided facilities for the clinical part of this work to be carried out in the Meyerstein Institute of Radiotherapy, Middlesex Hospital. Part of the expense of this investigation was defrayed by the British Empire Cancer Campaign.

### REFERENCES.

GLUCKSMANN, A.—(1947) 'Recent Advances in Clinical Pathology.' London (J. & A. Churchill, Ltd.), p. 338.

KOLLER, P. C.—(1948) *Brit. J. Radiol.*, Suppl. No. 1, p. 84.

*Idem* AND SMITHERS, D. W.—(1946) *Ibid.*, **19**, 89.

LEA, D. E.—(1947) 'The Actions of Radiations on Living Cells,' London (Cambridge University Press), p. 225.

PATERSON, R.—(1948) 'The Treatment of Malignant Disease by X-rays and Radium.' London (Edward Arnold & Co.), p. 171.

SPEAR, F. G. and GLUCKSMANN, A.—(1938) *Brit. J. Radiol.*, **11**, 533—(1939), *Ibid.*, **12**, 486.